# Secondary cancer in a survivor of Hodgkin’s lymphoma: A case report and review of the literature

**DOI:** 10.3892/ol.2014.2799

**Published:** 2014-12-12

**Authors:** MAJA LISIK-HABIB, URSZULA CZERNEK, SYLWIA DĘBSKA-SZMICH, MAGDALENA KRAKOWSKA, JOANNA KUBICKA-WOŁKOWSKA, PIOTR POTEMSKI

**Affiliations:** Department of Chemotherapy, Medical University of Lodz, Copernicus Memorial Hospital, Lodz 93-513, Poland

**Keywords:** Hodgkin’s lymphoma, secondary cancer, breast cancer, gastrointestinal cancer, cancer survivorship

## Abstract

Hodgkin’s lymphoma (HL) is one of the most curable malignant diseases in adults. However, HL patients have a higher risk of developing second malignancies compared with the general population. The population of adult cancer survivors is growing, thus, the long-term effects of cancer treatment, including secondary cancer development, have become an increasingly important concern in the field of oncology. The current study presents the case of a female HL survivor who developed two secondary malignancies within 29 years of follow-up. Furthermore, a review of the literature was conducted, which focused on secondary breast and gastrointestinal cancers in HL survivors.

## Introduction

Hodgkin’s lymphoma (HL), which predominantly occurs in young and middle-aged individuals, is one of the most curable malignant diseases in adults. Modern treatments have increased the number of malignant disease survivors, for example therapeutic advances over the past few decades have resulted in a significant improvement in the HL five-year survival rate (1,2). However, cancer survivors are at risk of developing secondary malignancies. Thus, late complications of cancer therapy, including secondary cancer development, are now an important area of concern. The current study presents the case of an HL survivor who was treated for HL in 1985 and developed two secondary malignancies within 29 years of follow-up. Written informed consent was obtained from the patient.

## Case report

### Hodgkin’s lymphoma

In June 1985, a 43-year-old, previously healthy female presented at the Regional Oncological Center of Copernicus Memorial Hospital (Lodz, Poland) with a two-week history of a painless enlargement of the left supraclavicular lymph nodes. No fever, pruritus, night sweats or weight loss were noted. Physical examination revealed hard, immovable, ~4 cm in diameter lymph nodes in the left supraclavicular area. Furthermore, bilateral hilar enlargement was identified upon chest X-ray. An excisional biopsy of the enlarged lymph node was performed and a diagnosis of HL (type, nodular sclerosis) was established. Additionally, a bone marrow biopsy and exploratory laparotomy were performed. The disease was staged at IIA according to the Ann Arbor staging system (3) and, in accordance with the standard of treatment at the present time, the patient underwent a splenectomy and received three cycles of MOPP/ABVE regimen chemotherapy (nitrogen mustard, 6 mg/m^2^ on day 1; vincristine, 2 mg on day 1; procarbazin, 100 mg/m^2^ on days 1–7; doxorubicin, 35 mg/m^2^ on day 8; bleomycin, 10 mg/m^2^ on day 8; vinblastine, 6 mg/m^2^ on day 8; prednisolone, 40 mg/m^2^ on days 1–14). After completion of the chemotherapy regimen, the patient was irradiated in the supradiaphragmatic and paraaortic lymph node areas, and achieved complete remission.

### Secondary breast cancer

In June 2009, a screening mammography revealed an irregular high-density mass (size, 45×50 mm) with clusters of microcalcifications and a Breast Imaging, Reporting and Data System (4) score of 5 in the upper outer quadrant of the right breast. A fine needle aspiration biopsy was performed and breast cancer was confirmed (i.e., cancer cells were detected). In July 2009, the patient underwent a radical right mastectomy with axillary lymphadenectomy. Subsequent histopathological examination determined an invasive, grade two, estrogen and progesterone receptor (ER and PR)-positive (score 3+), HER2-negative ductal carcinoma lesion of the breast (diameter, 2.5 cm) and metastasis to one of the axillary lymph nodes. Consequently, the patient received adjuvant treatment with tamoxifen (dose, 20 mg/day) and remained in remission.

### Secondary colon cancer

In April 2010 the patient began to complain of pain in the lower abdomen and persistent constipation. No obvious abnormalities were observed upon physical examination, however, morphological analysis revealed microcytic anemia: Red blood cells, 3.2 M/μl (normal range, 3.50–5.0 M/μl); hemoglobin, 10.2 g/dl (normal range, 11.2–15.0 g/dl); mean cell volume, 76.3 fl (normal range, 81–98 fl) and a high platelet count of 549 K/μl (normal range, 150–400 K/μl). The patient was referred for a colonoscopy, which identified a mass in the left colic flexure. A pathology report of the biopsied mass indicated adenocarcinoma, and chest and abdomen computed tomography (CT) scans revealed no distant metastasis. The patient subsequently underwent a left hemicolectomy. The pathology report determined moderately differentiated pT3 adenocarcinoma, according to TNM staging system (5), and 5/20 pericolic lymph nodes were positive for stage IIIB metastatic carcinoma. A standard FOLFOX-4 adjuvant chemotherapy commenced, however, oxaliplatin was discontinued after nine cycles due to grade two neurotoxicity. Adjuvant chemotherapy was completely terminated in December 2010 after 12 courses.

One year later, the patient presented with a persistent dry cough of one month. An antibiotic treatment was administered due to the suspicion of a protracted infection of the lower respiratory tract, however, no improvement was observed. A chest X-ray identified non-specific densities at the right infraclavicular area and a chest CT scan confirmed the presence of two metastatic lesions in the second segment of the right lung (13 and 5 mm in diameter). The patient underwent a videothoracoscopy with a radical wedge resection. The pathology report indicated grade two metastatic tubular adenocarcinoma, which possibly corresponded to colorectal cancer metastasis; however, a positron emission tomography-CT scan did not reveal any other metastatic lesions. Following surgery the patient received no systemic treatment and remains under surveillance.

## Discussion

Secondary malignancies are serious adverse effects of systemic therapy. According to the Surveillance, Epidemiology and End Results Program, secondary malignancies account for 16% of all cancer (6). Initially, the increased occurrence of secondary malignancies was reported in HL and multiple myeloma survivors (7,8). Subsequent malignancies are now the leading cause of mortality in HL survivors (9). Various studies have assessed or quantified the risk of developing secondary cancers following the successful treatment of HL (9–20), however these studies have not produced consistent results, possibly due to differences in the number of patients included in the analyses, the characteristics of the study population, the length of the follow-up period, the chemotherapy schedule, the age of HL diagnosis, and the dose and volume of irradiation. Prior radiotherapy is considered to be a major risk factor in the development of secondary malignancies. As a combined modality treatment of radiotherapy and chemotherapy was frequently used in previous decades, less is known about effects of chemotherapy alone (10). Breast cancer is the most frequent solid tumor and the leading cause of mortality among female HL survivors (11–15); this increased risk of breast cancer persists >30 years after the primary treatment of HL (21,22).

The incidence of secondary breast cancer depends on the dose and volume of irradiation, chemotherapy regimen used and the age at the time of treatment. The greatest risk of developing secondary breast cancer is reported in females who are <30 years old during HL treatment and in females administered with a treatment strategy of radiotherapy alone. The cumulative lifetime incidence in these females is almost 30% (23). Mantle field irradiation results in >2-fold increase in the risk of developing secondary breast cancer compared with the administration of a similar dose (36–44 Gy) of radiation restricted to the mediastinum (24). However, premature menopause and shorter exposure to endogenous estrogens caused by antineoplastic therapy appear to be protective factors (24–26). Furthermore, gonadotoxic treatment, which includes alkylating-based regimens and pelvic irradiation (dose, >5 Gy), results in a lower incidence of breast cancer (16,18). However, in a recent meta-analysis, Ibrahim *et al* (22) assessed the risk of HL survivors developing secondary breast cancer and could not demonstrate a protective effect of gonadotoxic alkylating chemotherapy against this development. Despite this, Ibrahim *et al* did demonstrate that the use of chemotherapy as the sole therapeutic modality was not associated with a significantly greater relative risk (RR) of developing breast cancer [RR, 1.19; 95% confidence interval (CI), 0.50–2.82].

Breast cancer in HL survivors with a history of radiation occurs at younger age compared with the general population. The breast cancer is typically bilateral and appears to be independent to other risk factors, such as obesity, smoking or the use of oral contraceptives (27–28). Secondary cancers are more often ER- and PR-negative when compared with primary tumors (27,29,30). Less is known regarding the development of other solid tumors in HL survivors, although there is an increased cumulative risk of developing solid cancers other than breast cancer compared with the general population. The most common sites of secondary and primary cancer development are similar (9,31–35). Hodgson *et al* (9) identified that males and females diagnosed with HL at the age of 30 years have an 18 and 26% thirty-year cumulative risk of developing secondary malignancies, respectively. Hodgson *et al* described 850 secondary cancers among 1,490 patients HL survivors and identified the colorectum as one of the predominant sites of the excess cancers (7% of all excess cancers). Furthermore, the HL survivors exhibited a similar absolute risk of colorectal cancer at 35–40 years compared with the general population at the age of 50–54 years old. Youn *et al* (34) assessed long-term survival among HL survivors who developed secondary gastrointestinal cancers and demonstrated that overall survival was significantly reduced in comparison to primary cancer patients. Although radiotherapy is the predominant risk factor for development of secondary cancers, the risk is also increased following chemotherapy alone. In a British study, Swerdlow *et al* (35) reported the RR of secondary cancers as 3.9 (95% CI, 3.5–4.4) after combined modality treatment and 2.0 (95% CI, 3.5–4.4) after chemotherapy alone. The risk of secondary cancers remained increased for >25 years after treatment. Furthermore, Swerdlow *et al* demonstrated that the 20-year cumulative risk of developing secondary cancers is 13% for patients treated with chemotherapy alone and 18% for patients who underwent combined modality treatment. Secondary malignancies were observed in 459/5,798 HL survivors, of which 27 (5.8%) were colorectal cancer. Combined-modality treated patients exhibited a significantly increased risk of developing colorectal cancer (RR, 2.0; 95% CI, 1.2–3.2; P<0.05) in comparison with chemotherapy alone, which was not associated with significant risk (RR, 1.1; 95% CI, 0.5–1.9). However, chemotherapy alone significantly increased the risk of developing lung cancer, leukemia and non-HL (35).

In conclusion, it is necessary to assess the potential risks and long-term complications of anticancer therapy among cancer survivors. Future efforts should be focused on developing novel screening strategies for individuals who have survived cancer, educating patients and introducing effective prevention strategies. Secondary malignancies appear at a younger age in cancer survivors compared with the general population, however, younger individuals are not recommended for routine screening. Thus, patients should be informed of the likelihood of developing secondary malignancies and preventative strategies among cancer survivors should be promoted.
